# Radiological informed consent in cardiovascular imaging: towards the medico-legal perfect storm?

**DOI:** 10.1186/1476-7120-5-35

**Published:** 2007-10-04

**Authors:** Gigliola Bedetti, Cosimo Loré

**Affiliations:** 1 Hospital S. Maria della Scaletta, Imola, Italy; 2 Institute of Legal Medicine, University of Siena, Italy

## Abstract

Use of radiation for medical examinations and tests is the largest manmade source of radiation exposure. No one can doubt the immense clinical and scientific benefits of imaging to the modern practice of medicine. Every radiological and nuclear medicine examination confers a definite (albeit low) long-term risk of cancer, but patients undergoing such examinations often receive no or inaccurate information about radiological dose exposure and corresponding risk directly related to the radiological dose received. Too detailed information on radiological dose and risk may result in undue anxiety, but information "economical with the truth" may violate basic patients' rights well embedded in ethics (Oviedo convention 1997) and law (97/43 Euratom Directive 1997). Informed consent is a procedure needed to establish a respectful and ethical relation between doctors and patients. Nevertheless, in an "ideal" consent process, the principle of patient autonomy in current radiological practice might be reinforced by making it mandatory to obtain explicit and transparent informed consent form for radiological examination with high exposure (≥ 500 chest x-rays). The form may spell-out the type of examination, the exposure in effective dose (mSv), derived from reference values in guidelines or – better – from actual values from their department. The dose equivalent might be also expressed in number of chest radiographs and the risk of cancer as number of extra cases in the exposed population, derived from most recent and authorative guidelines (e.g., BEIR VII Committee, release 2006). Common sense, deontological code, patients'rights, medical imaging guidelines, Euratom law, all coherently and concordantly encourage and recommend a justified, optimized, responsible and informed use of testing with ionizing radiation. Although the idea of informed consent for radiation dose does not seem to be on the immediate radar screen at least in the US, the current practice clashes against these guidelines and laws.

## Background

Every radiological and nuclear medicine examination confers a definite (albeit low) long-term risk of cancer, but patients undergoing such examinations often receive no or inaccurate information about these risks, directly related to the radiological dose received [1-5]. A too detailed information on radiological dose and risk may result in undue anxiety, but an information "economical with the truth" may violate basic patients' rights well embedded in ethics (Oviedo convention 1997) [6] and law (97/43 Euratom Directive 1997) [7]. In fact one of the three fundamental principles of the "charter of medical professionalism" in the new millennium is the principle of patient autonomy: "Physicians must empower their patients to make informed decisions about their treatment" [8]. The aims of this review are: 1 – to assess the information perceived by patients on radiation doses associated with common radiological procedures; 2- to review the physician awareness of doses (and risks) of examinations they daily prescribe and/or perform; 3- to discuss the ways this information is usually given to patients for exams regarding cardiovascular imaging; 4- to propose a better way to communicate radiation risk in keeping with modern medical guidelines, law prescription, deontological code and patients' rights; 5- to guard on the legal consequences on present medical policy on radiological risk.

### Patient's unawareness of radiological risk

Informed consent for radiological examinations is often not sought, and when it is, patients are often not fully informed, even for considerable levels of radiation exposure and long term risk [2]. This risk of a 64-slice computed tomography coronary angiography can be as high as 1 in 100 in a young woman or in a child [9]. In theory, the majority of pediatricians from the Greater Toronto Area, in Canada, practicing in a wide variety of hospital and clinical settings believe that a risk of 1 in 10,000 or more should be discussed with the parents [10]. In reality, patients are not given information about the risks, benefits, and radiation dose for a CT scan, even when a considerably higher risk is involved. In another study performed in the Emergency Department of a US academic medical center, adult patients who underwent diagnostic CT scan were surveyed. Only 7% of patients reported that they were told about risks of their CT scan, and all patients were unable to estimate the dose for one CT scan compared with that for one chest radiograph [10]. Only 3% of patients believed that their lifetime risk for cancer was increased as a result of the CT scan [10]. In another study performed in the Nuclear Medicine Department of a leading academic center in Italy, 79% of surveyed patients thought that the cardiac stress scintigraphy they had performed gave a radiation dose of <1 chest x-ray (instead of the true dose of 500 chest x-rays), and 40% thought that no cancer risk was present. Ironically, 71% of patients thought they received good-to-excellent informative on risks and benefits of the cardiac stress scintigraphy from the practicing physician [11].

### Physicians unawareness of radiological risk

Extensive recent data show substantial unawareness of radiological doses, and risks, not only of patients but of prescribing and practising doctors as well. In theory, good medical practice warrants knowledge of the doses and long-term risks of these tests – which can be judiciously employed when they are most appropriate. The results of surveys recently performed on British physicians [12], Israeli orthopaedists [13], Italian cardiologists [14], Canadian pediatricians [15], and US academic radiologists [15], show that the majority of doctors grossly underestimate the radiation doses (usually by up to 500 times) and corresponding cancer risks for most commonly requested investigations. Emergency Room physicians, and radiologists alike are unable to provide accurate estimates of CT doses regardless of their experience level. In particular, among radiologists, 5% of respondents thought that a computed tomography scan dose was less than one chest radiograph, and 56% estimated the computed tomography scan dose between 1 and 10 chest radiographs, with dramatic underestimation of the true dose (about 500 chest radiographs) [15]. Forty percent of pediatricians underestimate of up to 100 times the dose of a pre- and post-contrast head CT [10]. A minority of doctors also suffers of what we might call "imaging daltonism", i.e. the inability to separate "green" (non-ionizing) from "red" (ionizing) techniques. Five percent of British doctors does not realise that ultrasound does not use ionizing radiation, and 10% does not realise that magnetic resonance imaging does not use ionizing radiation [12]. Among Canadian paediatricians, 4% believed Ultrasound involves ionizing radiation and 12% did not appreciated that scintigraphy scans do [10]. In presence of this diffuse background level of radiological unawareness, inappropriate examinations may proliferate with deep potential societal and patients' detriment [16-19].

### Informed consent: how it is

There are three possible ways to look at radiologic risk communication in medicine – no mention of risk, understatement of risks, and specific detailing of risks [2].

#### Strategy 1: "Don't say a word"

One philosophy is not to mention radiological risk. Even for procedures with high radiation dose, such as interventions under fluoroscopic control, there is no explicit or implicit mention of long term risks. The risk exists and may be substantial, but it remains unheard (by the patient) and unspoken (by the doctor). The basic argument is that radiologists are too busy to loose time in obtaining informed consent and too wise to undertake inappropriate examinations [20]. Patients' legal right to information is eclipsed by the two forces of efficiency and a paternalistic, "expert knows best" vision of individual autonomy. The long term nature of the risk, not its absolute amount, seems to be the excuse for overlooking the issue of informed consent.

#### Strategy 2: Understatement

In other aspects of radiological practice obtaining written informed consent is part of standard practice. In this case, the issue of efficiency bias is not raised: a patient must give informed consent before contrast is injected. But what is the quality of the information given to patients? On the websites of scientific societies, in the information section for patients and in the informed consent forms to be signed by patients, we read statements such as for instance: "A nuclear medicine examination is safe, with an irradiation corresponding to a simple radiograph" or "almost always less than a common radiological examination"[20]. Both patients and clinicians might believe that a "common radiological examination" or "a simple radiograph" would be a chest x ray, which is by far the simplest and commonest radiological examination [21]. In reality, however, the dose exposure ranges in cardiology from 500 chest x-rays for a sestamibi to 1500 chest x-rays for a dual isotope cardiac stress scintigraphy [22]. Such imprecise statements are probably intended to reassure patients, to avoid useless concern about an unavoidable risk. However, this attitude of "one consent fits all" for radiological examinations may mislead clinicians to underestimate the associated risks.

#### Strategy 3: Full disclosure

Some organisations, such as the US National Institutes of Health, describe radiological risk in more straightforward terms, at least when the test is performed within a research project and with a radiation dose greater than 15 millisieverts (corresponding to the average dose of 64-slice computed tomography coronary angiography): "Your scan involves exposure to radiation. Although it can vary from person to person, your whole body radiation exposure during each scan will be about 15 millisieverts. This is about five times the average annual radiation exposure a person in the United States receives from natural background radiation. Although no harmful effects are expected, your long term risks of harm from this degree of radiation exposure might be as high as 1 in 1000. Harmful effects could include the development of cancer and genetic changes" [23].

### Informed consent: how it should be

Non-specialists (and sometimes specialists) often do not understand the difficult jargon of radiation protection, in which doses are expressed in many varied units (megaBecquerel, milliCuries, kilovolts, dose-area product, etc), and simple information on doses and risks is difficult to find and hard to interpret [2]. The pressures of an old-fashioned paternalistic view of medicine and of a more modern efficientism act against the building of a really informed consent [2].

**Figure 1:**
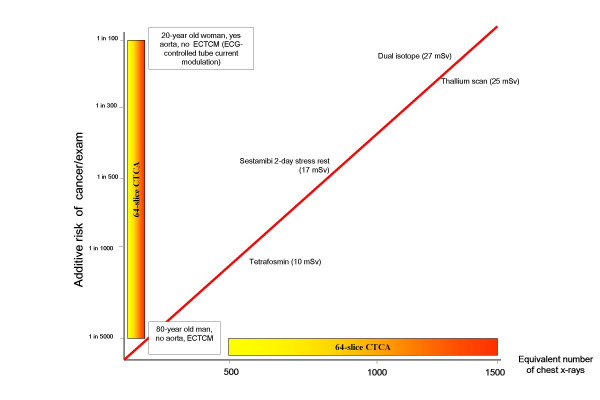
Dose (in x-axis, in equivalent dose in chest x-rays) and risks (in y-axis, calculated from BEIR VII) of commonly performed examinations. Abbreviations: CTCA, computed tomography coronary angiography; ECTCM, electrocardiographically controlled tube current modulation.

Nevertheless, in an "ideal" consent process, the principle of patient autonomy in current radiological practice might be reinforced. In our opinion, a formal informed consent should be obtained for procedures involving high radiation doses (let's say, ≥ 500 chest x-rays) and we believe certain information should be included on the form [2]. There is no doubt about the different ethical basis when participants are irradiated for purely research purposes, with no prospect of personal benefit, compared with diagnostic tests for patients (screening has a broadly intermediate ethical position). This is clearly a justification for a more explicit approach to obtaining informed consent. The standard of risk communication already adopted for irradiation in research might be fruitfully followed for irradiation in clinical practice. The form should spell-out at least the type of examination, the exposure in effective dose (mSv), the dose equivalent in number of chest radiographs, and the risk of cancer as number of extra cases in the exposed population [1,2,5]. Obviously, doses and risks vary widely according to the type of technology and types of imaging scan protocol. The risk for the same physical dose varies according to age and gender [3,4]. Table 1 reports an example applied to 4 types of stress perfusion. Ideally, all institutions would be required to produce effective doses for procedures in their department, which may not be directly equivalent to those reported in the literature [16-19]. Table 1 shows the wide range of doses, and risks, for a very common 64-slice Computed tomography coronary angiography, as recently proposed by Bedetti et al [11]. The dose can increase three-fold if the scan is performed without electrocardiographically controlled tube current modulation and extending the baseline scan by 10 cm cranially to include the aortic arch. The risk increases with younger age and female gender, and can be as low as 1 in 5017 in a 80-year-old man with scan not including aorta and performed with electrocardiographically- controlled tube current modulation, and as high as 1 in 114 for a 20-year-old woman with a combined scan of the heart and aorta without electrocardiographically controlled tube current modulation [9]. The associated graph (Figure 1) underlines the linear relation between dose and risk and might be useful for passing information from doctors to patients and between doctors because the figure format is more easily understood than the traditional table format and the colour coding helps readers to understand risk levels [2]. As a reference, Figure 1 shows also 4 types of cardiac stress scintigraphy perfusion imaging with 4 different protocols: Tc-99m tetrofosmin rest stress (10 mSv); Tc-99m sestamibi 2-day stress rest (17 mSv); Tl-201 stress and reinjection (25 mSv); Dual-isotope (Tl-201 and Tc-99m) stress imaging (27 mSv) [22]. The clinical information provided by the 4 protocols is basically the same, and can be obtained with no radiation exposure by ultrasound or magnetic resonance imaging [24]. This simple, evidence based communication strategy, if used when obtaining informed consent, will increase the currently suboptimal level of radiological awareness among doctors and patients. Better knowledge of risks will help us to avoid small individual risks translating into substantial population risks [16-19]. Consent forms would also help reduce pressure from patients for redundant and often useless examinations [2].

**Table 1:** Ways to communicate risk

**64-slice Computed Tomography Coronary Angiography**	**Effective Radiation Dose (mSv)**	**Equivalent Number of chest radiographs**	**Lifetime additional risk of cancer**
No aorta, yes ECTCM, 80-year old man	9	450	1 in 5017
Yes aorta, no ECTCM, 20-year old woman	29	1450	1 in 114

ECTCM, electrocardiographically controlled tube current modulation. Data from ref. 9, Einstein et al.

### Radiological informed consent : towards the perfect legal storm

Patients undergoing common imaging examinations involving significant exposure have little or no awareness about radiological dose exposure (and corresponding risk). This ineffective communication poses significant ethical problems, with high litigation potential. Informed consent is a procedure needed to establish a respectful and ethical relation between doctors and patients. Use of radiation for medical examinations and tests is the largest manmade source of radiation exposure [1]. Small individual risks of each test performed with ionizing radiation multiplied by billions of examinations become significant population risks. For this reason, in Europe both the law [7] and the Referral Guidelines for Medical Imaging [17] recommend a justified, optimized and responsible use of testing with ionizing radiation. The Euratom Directive 97/43 establishes that the indication and execution of diagnostic procedures with ionizing radiation should follow three basic principles: the justification principle (article 3: "if an exposure cannot be justified, it should be prohibited"); the optimization principle (article 4: according to ALARA principle, "all doses due to medical exposures must be kept As Low As Reasonably Achievable") and the responsibility principle (article 5: "both the prescriber and the practitioner are responsible for the justification of the test exposing the patient to ionizing radiation") [7]. European Commission referral guidelines were released on 2001 in application of Euratom Directive and evolved from those previously published by the UK Royal College of Radiology in 1998 [16]. They explicitly state that a non-ionizing technique must be used whenever it will give grossly comparable information to an ionizing investigation. For instance, "because MRI does not use ionizing radiation, MRI should be preferred when both CT and MRI would provide similar information and when both are available" [17]. Ultrasound or MRI should be the preferred option for assessing cardiac function or myocardial ischemia, when long-term risks are included in the decision-making [4]. However, in spite of the existing European law and European Commission recommendations they are not so strictly reinforced, and at least 30% of all ionizing testing procedures remain inappropriate in clinical practice [25,26]. Common sense, deontological code, patients' rights, medical imaging guidelines, Euratom law, all coherently and concordantly suggest, encourage and order a responsible and informed use of ionising testing. The current practice clashes against these guidelines and laws [24,25]. It will become more and more difficult to defend physicians ignoring doses and risks of exams with high radiation load, especially in case of inappropriate examinations, which plague – in all fields – the current practice of medicine [26-28].

## Abbreviations

ICRP = International Commission on Radiological Protection, BEIR VII = Biologic Effects of Ionizing Radiation VII.

## Competing interests

The author(s) declare that they have no competing interests.

## Authors' contributions

GB and CL participated to the writing of the manuscript. All authors read and approved the final manuscript.
